# An Smp43-Derived Short-Chain α-Helical Peptide Displays a Unique Sequence and Possesses Antimicrobial Activity against Both Gram-Positive and Gram-Negative Bacteria

**DOI:** 10.3390/toxins13050343

**Published:** 2021-05-11

**Authors:** Xudong Luo, Li Ding, Xiangdong Ye, Wen Zhu, Kaiyue Zhang, Fangyan Li, Huiwen Jiang, Zhiwen Zhao, Zongyun Chen

**Affiliations:** 1 Department of Biochemistry and Molecular Biology, Institute of Basic Medical Sciences, College of Basic Medicine, Hubei University of Medicine, Shiyan 442000, China; luoxudong000@126.com (X.L.); dl2168@163.com (L.D.); yexiangdong7237@163.com (X.Y.); zhuwen0712@126.com (W.Z.); zky512@foxmail.com (K.Z.); lifangyan000@sina.com (F.L.); jhw0908@163.com (H.J.); zhaozw1999@126.com (Z.Z.); 2 Hubei Key Laboratory of Wudang Local Chinese Medicine Research, Hubei University of Medicine, Shiyan 442000, China; 3 Department of Clinical Laboratory, Dongfeng Hospital, Hubei University of Medicine, Shiyan 442000, China

**Keywords:** scorpion venom, noncysteine-containing AMPs, Smp43, Smp43(1-14), antimicrobial resistance, HeliQuest

## Abstract

Scorpion venoms are rich resources of antimicrobial peptides (AMPs). While the short-chain noncysteine-containing AMPs have attracted much attention as templates for drug development, the antimicrobial potential of long-chain noncysteine-containing AMPs has been largely overlooked. Here, by using the online HeliQuest server, we designed and analyzed a series of 14-residue fragments of Smp43, a 43-residue long-chain noncysteine-containing AMP identified from the venom of *Scorpio maurus palmatus*. We found that Smp43(1-14) shows high antimicrobial activity against both Gram-positive and Gram-negative bacteria and is nontoxic to mammalian cells at the antimicrobial dosage. Sequence alignments showed that the designed Smp43(1-14) displays a unique primary structure that is different from other natural short-chain noncysteine-containing AMPs from scorpions, such as Uy17, Uy192 and IsCT. Moreover, the peptide Smp43(1-14) caused concentration-dependent fluorescence increases in the bacteria for all of the tested dyes, propidium iodide, SYTOX^TM^ Green and DiSC_3_-5, suggesting that the peptide may kill the bacteria through the formation of pore structures in the plasma membrane. Taken together, our work sheds light on a new avenue for the design of novel short-chain noncysteine-containing AMPs and provides a good peptide template with a unique sequence for the development of novel drugs for use against bacterial infectious diseases.

## 1. Introduction

Scorpions are regarded as some of the oldest and most widely distributed animals on the planet. Over the long-term, through survival pressure and the evolutionary adaptation process, scorpions have built up their abilities of predation and defense through the venom toxin secreted from their venom glands [1,2]. It has been reported that some of the most important components of scorpion venom are peptides [3]. Scorpion venom derived peptides display various biological activities that include ion (Na^+^, K^+^, Cl^+^ and Ca^2+^) channel modulation [4,5,6,7,8], protease inhibition [9,10,11], anticoagulation [12], immunomodulation [13], anticancer activity [14] and antimicrobial activity [15]. These peptides are a rich resource for the development of drug leads with a wide range of therapeutic applications [16].

Dozens of antimicrobial peptides (AMPs) have been identified from the venom glands of various scorpion species. These peptides are generally divided into two groups. Cysteine-containing AMPs, also known as defensins, contain three or four disulfide bridges in their structures and usually form a cysteine-stabilized α-helix and β-sheet fold [17]. The majority of scorpion venom-derived AMPs are noncysteine-containing AMPs. These noncysteine-containing AMPs consist of 13–56 amino acids and can be further divided into three subfamilies according to their sequence length: short-chain peptides (< 20 amino acids), intermediate-chain peptides (20–35 amino acids) and long-chain peptides (>35 amino acids) [17]. The intermediate-chain noncysteine-containing AMP family includes Im5 [18], pandinin-2 [19], meucin-24, meucin-25 [20], heterin-2 [21] and HsAP [22]. The short-chain noncysteine-containing AMP family includes a large number of peptides that consist of 13 or 14 residues, such as IsCTs [23,24], StCTs [15,25], BmKn2 [26], lausporin-1 [27], VmCTs, VsCTs [28], OcyCs [29], meucin-13 [30], Uy17, Uy192, UyCTs [31,32] and pantinins [33]. Owing to their small size, short-chain peptides are considered good templates for peptide-based drug design [26,34,35]. The long-chain noncysteine-containing AMPs from scorpion venoms are also an important natural peptide resource and include hadrurin [36], BmKbpp [37], opistoporins, parabutoporin [38], pandinin-1 [19], Im1 [39], vejovine [40] and Smp43 [41]. However, owing to their large size and high production cost, the antimicrobial application of long-chain noncysteine-containing AMPs has been largely overlooked. 

Smp43, identified from the venom gland of *Scorpio maurus palmatus*, is a 43-residue peptide that exhibits poor antimicrobial activity [41]. In this study, by analyzing a series of 14 residue fragments derived from Smp43, we found that Smp43(1-14), the N-terminal 14-residue fragment of Smp43, displays a unique sequence and shows antimicrobial activity against both Gram-positive and Gram-negative bacteria with minimum inhibitory concentrations (MICs) of 5–10 μg/mL, and is nontoxic to mammalian cells at the antimicrobial dosage. Moreover, Smp43(1-14) induced concentration-dependent fluorescence increases in the bacteria for all of the tested dyes, propidium iodide, SYTOX^TM^ Green and DiSC_3_-5, suggesting that the peptide may kill the bacteria through the formation of pore structures in the plasma membrane. Our work sheds light on a new avenue for the design of novel short AMPs and the peptide provides a good template for the development of novel antimicrobial agents. 

## 2. Results

### 2.1. Design of Short-Chain Peptides from the Long-Chain Noncysteine-Containing Peptide Smp43

**Figure 1:**
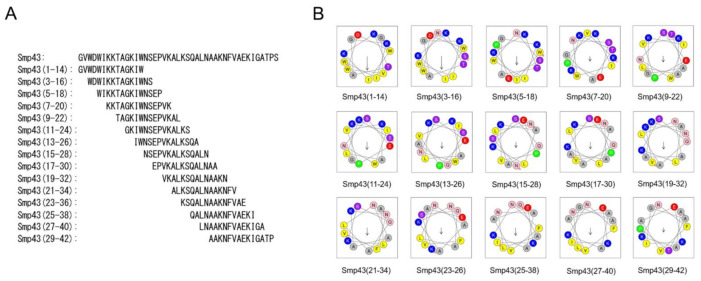
Design of short-chain antimicrobial peptides from scorpion venom-derived long-chain noncysteine-containing peptide. (**A**) Sequences of Smp43 derived short-chain peptides; (**B**) Helical wheel projections of the designed peptides. The alkaline and hydrophobic residues were shown in blue and yellow, respectively.

Long-chain noncysteine-containing AMPs from scorpion venoms are an important natural peptide resource (Table S1). However, their antimicrobial potential is largely unknown due to their large size and high cost of production. The aim of this study was to investigate whether effective antimicrobial peptides can be designed from the long-chain noncysteine-containing AMPs. For this purpose, Smp43 was selected for study. The online HeliQuest server offers a useful tool for antimicrobial peptide design and analysis [42]. By using this tool, a series of 14-residue peptides from Smp43 were obtained, and the peptides selected for further functional investigations are shown in Figure 1A and Table S2. All of the selected peptides were synthesized by solid-phase methods and, consistent with most natural short scorpion-derived AMPs [17], all of the synthesized short-chain peptides were also amidated at the C-terminus. Their molecular weights were measured by mass spectrometry (Table S2). Along with the design of the short-chain peptides, several parameters were generated. As shown in Table S2, all of the peptides have a net charge ranging from +2 to +4, indicating the cationic characteristics of these peptides. Smp43(1-14) has a mean hydrophobic value of 0.600, and a hydrophobic moment of 0.590, indicating moderate hydrophobicity and amphiphilicity (Table S2). These characteristics were further demonstrated by the helical wheel plot of Smp43(1-14) in which the nonpolar residues (Trp, Ile and Val) were positioned on one side of the peptide and most of the polar residues were positioned on the other side (Figure 1B). Compared with Smp43(1-14), Smp43(3-16) displays slightly lower amphiphilicity (0.572) but much lower hydrophobicity (0.467), and the residue arrangements in the helical wheel plot of this peptide differ greatly from those of Smp43(1-14) (Figure 1B and Table S2). Other peptides have much lower hydrophobicity and hydrophobic moment values and display staggered distributions of polar and non-polar amino acids (Figure 1B and Table S2), suggesting that these are not amphiphilic peptides. 

### 2.2. Smp43(1-14) Shows Antimicrobial Activity against Both Gram-Positive and Gram-Negative Bacteria

Bacteria resistant to traditional antibiotics have become a serious threat to human health, and novel antimicrobial agents are urgently needed. To evaluate the antimicrobial potential of the designed peptides, MIC determination was performed against several bacterial strains including Gram-positive bacteria *Staphylococcus aureus* and *Enterococcus faecalis*, and Gram-negative bacteria *Escherichia coli*, *Klebsiella pneumoniae*, *Pseudomonas aeruginosa* and *Acinetobacter baumannii*. These bacterial species have been reported to cause the majority of infections involved in antibiotic resistance [43,44]. As shown in Table 1, the MIC values of Smp43(1-14) against these bacterial species were determined to be 5–10 μg/mL, and, compared with other bacterial species tested, Smp43(1-14) was more potent against *S. aureus* and *A. baumannii* (MICs: 5 μg/mL). In contrast, the other synthesized peptides were inactive against all of the bacterial strains tested (Table S3).

**Figure 2:**
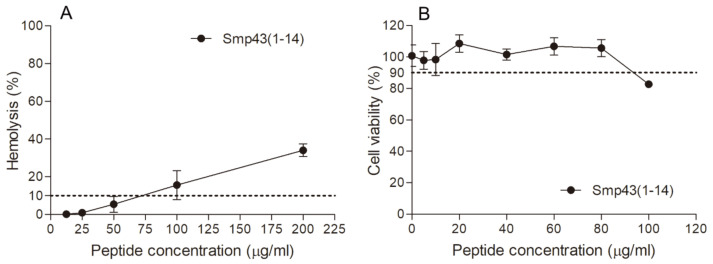
Hemolytic activity and cytotoxicity of Smp43(1-14). (**A**) The hemolytic activities of Smp43(1-14) at different concentrations were determined against human red blood cells. The absorbance of the supernatants was measured at 540 nm to evaluate the release of hemoglobin. (**B**) The cytotoxicity of the peptide was determined against L929 cells by measuring 2-(2-methoxy-4-nitrophenyl)-3-(4-nitrophenyl)-5-(2,4-disulfophenyl)-2H-tetrazolium (CCK-8) absorbance at 450 nm. The data shown are average ± standard error of three independent experiments.

The hemolytic activities of the designed peptides were measured against human red blood cells. Smp43(1-14) showed low hemolytic activity, as the peptide concentration that caused 10% hemolysis (HC_10_) was determined to be 75 μg/mL (Table 1 and Figure 2). The HC_10_ values of other peptides were not detected within the peptide concentration range tested (Table S3). To further evaluate the safety of Smp43(1-14), the cytotoxicity of the peptide was determined against mouse fibroblast cell line L929. As shown in Table 1 and Figure 2, the peptide concentration that caused 10% inhibition of cell growth was determined to be 92 μg/mL. Taken together, these results suggest that Smp43(1-14) is a peptide with high antimicrobial activity and low cytotoxicity.

### 2.3. Smp43(1-14) Is a Cationic Amphiphilic α-Helical AMP with a Unique Primary Sequence

**Figure 3:**
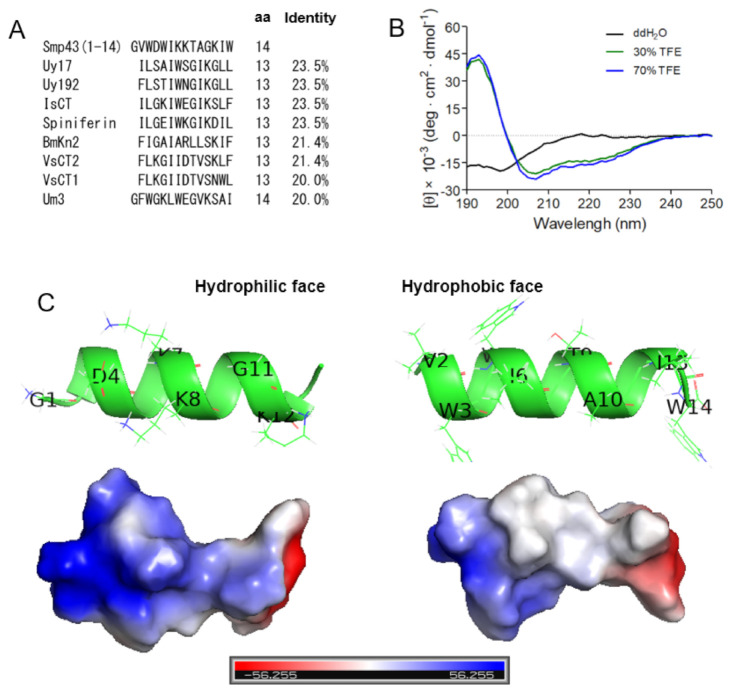
Sequence alignments and structural characterizations of Smp43(1-14). (**A**) Sequence alignments of Smp43(1-14) with other antimicrobial peptides were performed by using DNAMAN. (**B**) Far-ultraviolet CD spectra of Smp43(1-14). The scans were repeated three times for each curve. (**C**) The residual composition of the hydrophilic face (left panel) and the hydrophobic face (right panel) of Smp43(1-14). The three-dimensional structure was projected online by using the I-TASSER Server (https://zhanglab.dcmb.med.umich.edu/I-TASSER/, accessed on 13 April 2021).

Through testing a series of 14-residue fragments of Smp43, we identified Smp43(1-14) as a novel short-chain antimicrobial peptide. To investigate whether Smp43(1-14) has a unique primary sequence, sequence alignments were performed between Smp43(1-14) and other scorpion venom-derived short-chain noncysteine-containing peptides. As shown in Figure 3A and Table S4, Smp43(1-14) displayed low identity to all of the tested scorpion venom-derived AMPs, including Uy17 (23.5%), Uy192 (23.5%), BmKn2 (21.4%), Smp13 (17.7%) and Hp1404 (14.3%), indicating that Smp43(1-14) has a unique primary sequence.

To evaluate the structural characteristics of Smp43(1-14), circular dichroism (CD) spectra were measured in either ddH_2_O or 2,2,2-trifluoroethanol (TFE) solutions. As shown in Figure 3B, Smp43(1-14) exhibits a negative peak at approximately 200 nm in ddH_2_O, indicating a random coiled structure in aqueous solution, while the peptide exhibits a large positive peak at approximately 195 nm and two negative bands centered at 208 and 222 nm in TFE solutions, indicating that the peptide could form an α-helix-rich content in an appropriate membrane environment. The structural characteristics of Smp43(1-14) were also projected by using the I-TASSER server [45]. The peptide adopted an α-helical conformation, the basic residues (Lys7, Lys8 and Lys12) were gathered on the hydrophilic face of the α-helix and the peptide was more positively charged on this face; the hydrophobic residues (Val2, Trp3, Trp5, Ile6, Ile13 and Trp14) were positioned on the other face of the α-helix (Figure 3C). Taken together, these data suggest that Smp43(1-14) is a cationic amphiphilic α-helical AMP with a unique primary sequence. 

### 2.4. Antimicrobial Mechanism of Smp43(1-14)

**Figure 4:**
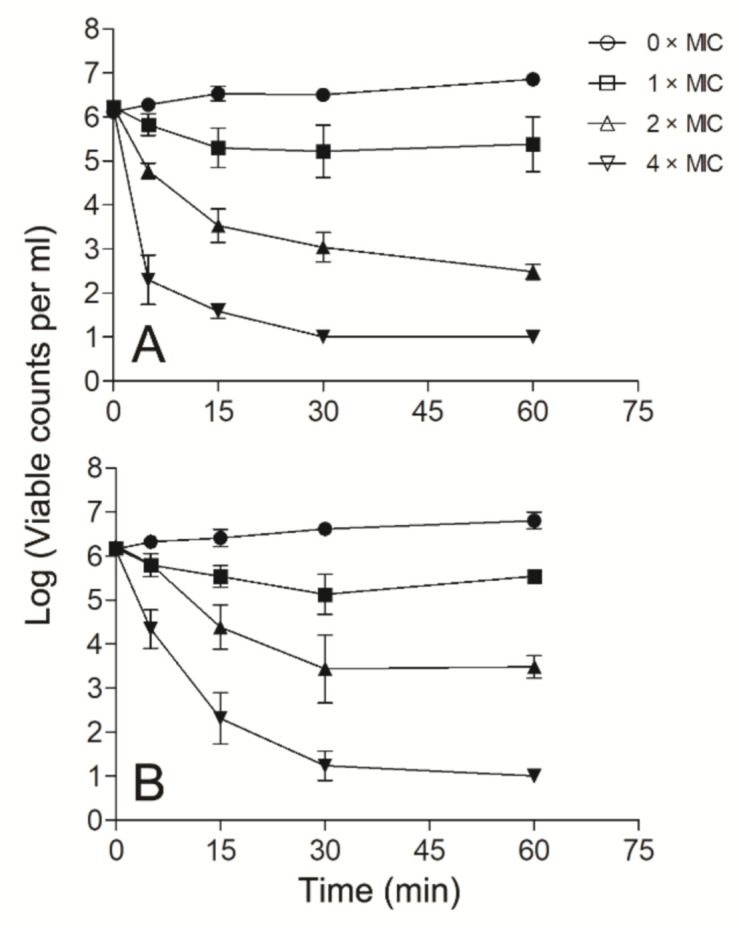
Time–killing kinetics of Smp43(1-14). The time–killing kinetics were determined against *S. aureus* ATCC29213 (**A**) and *A. baumannii* ATCC19606 (**B**). The peptide concentrations were 0 × MIC (circle), 1 × MIC (square), 2 × MIC (upward triangle) and 4 × MIC (downward triangle). The curves shown are average ± standard error of the data derived from three independent experiments.

**Figure 5:**
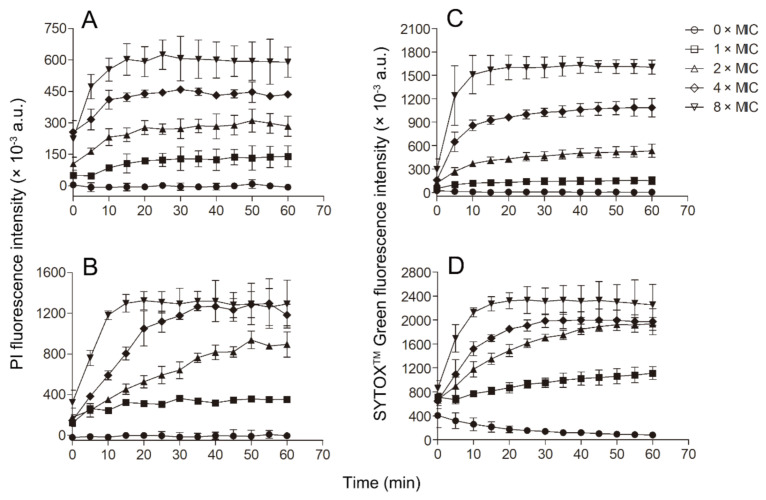
Membrane permeabilization of the bacterial cells caused by Smp43(1-14). (**A**,**B**) PI uptake assays; (**C**,**D**) SYTOX^TM^ Green uptake assays. (**A**,**C**) *S. aureus* ATCC29213; (**B**,**D**) *A. baumannii* ATCC19606. The concentrations of Smp43(1-14) were 0 × MIC (circle), 1 × MIC (square), 2 × MIC (upward triangle), 4 × MIC (diamond) and 8 × MIC (downward triangle). The curves shown represent the average ± standard error of three independent experiments.

Since Smp43(1-14) exhibited better antimicrobial activity against *S. aureus* and *A. baumannii* than other bacteria tested, further experiments were performed to evaluate the mode of action of Smp43(1-14) against these two pathogens. Smp43(1-14) showed a concentration-dependent bacterial killing kinetics against *S. aureus* ATCC29213 (Figure 4A). A similar phenomenon was observed in Smp43(1-14)-treated *A. baumannii* ATCC19606 (Figure 4B). The time-killing curves of Smp43(1-14) for the bacterial strains resemble those of membrane-targeting antibiotics, which exert their bactericidal activity by pore structure formation in cytoplasmic membrane of bacterial cells [46]; therefore, several fluorescence-based assays were performed to evaluate the effects of Smp43(1-14) on the plasma membrane of the two bacteria. Propidium iodide (PI) is a nucleic acid binding molecule that can permeate only compromised plasma membranes; accordingly, this probe has been used to investigate AMP-induced pore formation in bacterial membrane [47]. As shown in Figure 5A,B, upon the addition of Smp43(1-14), concentration-dependent PI fluorescence increases were observed in both *S. aureus* and *A. baumannii* samples, indicating that the peptide integrated with the plasma membrane and induced the formation of pore structures. This phenomenon was confirmed by using SYTOX^TM^ Green, where the molecule displayed similar nucleic acid binding ability but showed distinct spectroscopic properties [48]. The addition of Smp43(1-14) also led to dose-dependent SYTOX^TM^ Green fluorescence increases in these two bacterial species (Figure 5C,D).

**Figure 6:**
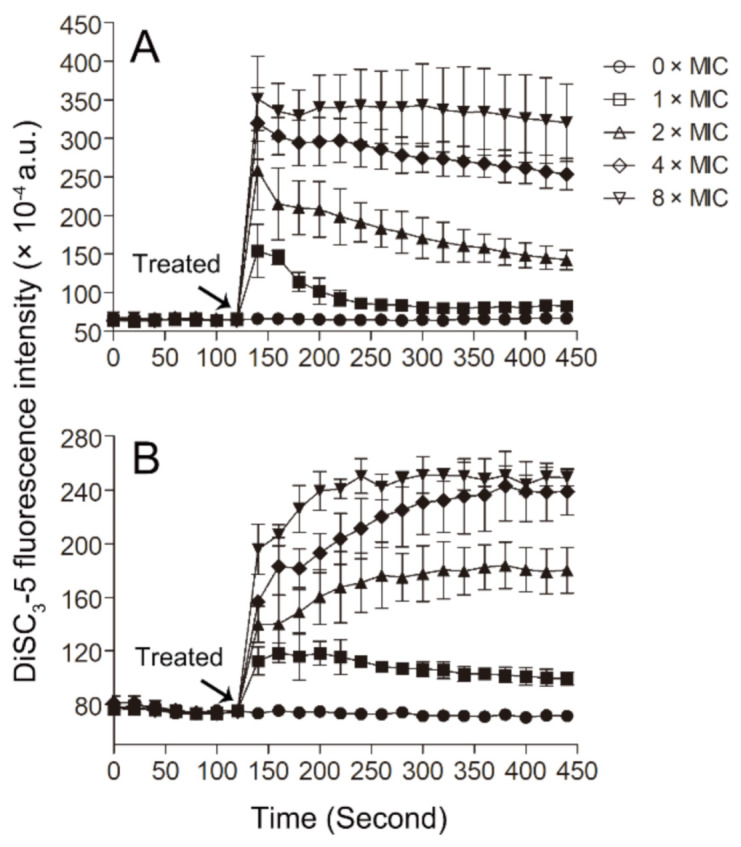
DiSC_3_-5 fluorescence assay. The changes of membrane potential of *S. aureus* ATCC29213 cells (**A**) and *A. baumannii* ATCC19606 cells (**B**) induced by Smp43(1-14) were monitored by measuring DiSC_3_-5 fluorescence. The concentrations of Smp43(1-14) were 0 × MIC (circle), 1 × MIC (square), 2 × MIC (upward triangle), 4 × MIC (diamond) and 8 × MIC (downward triangle). The data shown represent the average ± standard error derived from three independent experiments.

The probe 3,3′-dipropylthiadicarbocyanine iodide (DiSC_3_-5) has been widely used to monitor the disturbance of the membrane potential of the bacterial membrane. This probe can be inserted into the cytoplasmic membrane under quiescent conditions, and when the membrane potential is disturbed, the probe can be released into the solution and lead to an increase in fluorescence [49,50]. In our experiments, we found that Smp43(1-14) can induce membrane potential disturbance, as indicated by the dose-dependent increases in DiSC_3_-5 fluorescence in the bacterial samples upon the addition of the peptide (Figure 6). As the pore structures formed by the peptide can result in membrane potential disturbance, so, together with the PI and SYTOX^TM^ Green experiments, the results of DiSC_3_-5 experiments suggest that the peptide Smp43(1-14) may kill bacteria through the formation of pore structures in the plasma membrane of the bacteria. 

## 3. Discussion

Antibiotic resistance has become an increasing threat to human health. Antimicrobial peptides have attracted much attention as one of the promising solutions in the post-antibiotic era [51,52]. AMPs are small, positively charged, amphiphilic peptides with direct antimicrobial activity against a wide range of microbes, including viruses, bacteria, fungi and parasites [53]. Their antimicrobial mechanism typically starts with the electrostatic binding of the peptide to a negatively charged bacterial membrane, followed by the insertion of the peptide into the plasma membrane and the formation of various pores, which leads to bacterial cell death. From this mode of action, it has been reported that AMPs can kill bacteria with a much lower occurrence of the development of resistance [54,55,56,57]. In this work, by using the HeliQuest server, we screened and investigated a series of Smp43-derived 14-residue peptides and identified Smp43(1-14) as a novel antimicrobial peptide that is active against several Gram-positive and Gram-negative bacteria of important clinical significance. A mechanistic study suggests that Smp43(1-14) can bind to the bacterial membrane and may kill the bacteria by inducing pore structure formation of the plasma membrane.

The HeliQuest server has been widely used to calculate net charge, the mean hydrophobicity value and the hydrophobic moment of peptides, and to produce helical wheel plots, a plane projection drawing that presents the relative positions of the residues of an α-helical antimicrobial peptide [42]. However, it has almost never been reported that this platform can be used to screen peptides of special length from a long-chain peptide. In this work, a series of 14-residue peptides were generated from Smp43, accompanied by their physiochemical properties. Antimicrobial assays demonstrated that Smp43(1-14) showed good antimicrobial activity. More importantly, the convenience of this platform is that, as the peptide sequence was generated, several important parameters were also generated and could be readily analyzed. It has been reported that the net charge is helpful for the initial binding of a peptide to a negatively charged bacterial membrane [58,59,60]. Hydrophobicity is important for the interactions of peptides with membrane lipids [61,62,63,64,65]. Amphipathicity, which is indicated with a hydrophobic moment, is essential for the formation of various pores in membranes, such as barrel-stave pores, toroidal pores and peptide carpets [66,67,68,69]. Accordingly, the ineffectiveness of the other generated peptides can be explained by the low hydrophobicity and hydrophobic moment values displayed by these peptides. Therefore, our work suggests that the HeliQuest server can be a good tool for the identification of short-chain antimicrobial peptides from long-chain peptides or proteins. 

To date, many AMPs have been identified from a wide range of scorpion species. Most of these peptides were discovered through cDNA screening or transcriptome analysis after the identification of the first AMPs; therefore, the AMPs of similar sequence length showed relatively high sequence similarity. For example, the sequence identities between pantinin-1, -2 and -3 and other scorpion-derived antimicrobial peptides are higher than 50%, 60% and 60%, respectively [33]. In another study, Hp1404 was found to show high similarity (>64%) to other tested scorpion AMPs [27]. Here, Smp43(1-14) showed very low identity to all of the other scorpion-derived AMPs, such as IsCTs [23,24], StCTs [15,25], BmKn2 [26], Hp1404 [27], VmCTs, VsCTs [28], OcyCs [29], meucin-13 [30], Uy17, Uy192, UyCTs [31,32] and pantinins [33]. Our work provides a new short-chain noncysteine-containing AMP template, Smp43(1-14), with a unique sequence for further peptide design and optimization.

Excitingly, the natural long-chain noncysteine-containing AMP Smp43 is a 43-residue antimicrobial peptide identified from the venom gland of *Scorpio maurus palmatus*, which was reported to display poor antimicrobial activities to the tested pathogens [41]; however, the antimicrobial activities of Smp43(1-14) to clinically relevant bacterial species were significantly improved compared with those of the parent peptide. Smp43(1-14) shows antimicrobial activity against several pathogens for which novel antimicrobials are urgently needed, and is non-toxic at the antimicrobial dosage. The broad-spectrum antimicrobial activity, good safety, small size and unique primary sequence make it a good starting point for peptide drug development. Overall, our work sheds light on the experimental conversion of a scorpion-derived peptide from the long-chain noncysteine-containing AMP family into a short-chain noncysteine-containing AMP with effective antimicrobial activity. 

## 4. Materials and Methods

### 4.1. Peptide Design, Analysis and Synthesis

The peptides studied in this work were designed by using the online HeliQuest server (https://heliquest.ipmc.cnrs.fr/, accessed on 13 April 2021) [42]. The parameters were set as follows: ‘Helix type’ was set as ‘α’ and ‘Window size’ was set as ‘14’. The peptides selected for investigation were synthesized by Sangon Biotech (Shanghai, China) by using solid-phase methods based on standard N-9-fluorenylmethyloxycarbonyl (Fmoc) chemistry [70]. The purities of the peptides (>95%) were determined by reverse-phase HPLC. The molecular weights of the peptides were confirmed by mass spectrometry. Sequence alignments were performed by using DNAMAN [71]. The helical-wheel plots, net charge, mean hydrophobic value and hydrophobic moment were obtained by using the HeliQuest server [42]. The three-dimensional structures were projected by using the online server I-TASSER (https://zhanglab.dcmb.med.umich.edu/I-TASSER/, accessed on 13 April 2021) [45].

### 4.2. CD Spectroscopy

The CD spectra (λ_190−250 nm_) were measured on a J-820 spectropolarimeter (Jasco, Tokyo, Japan). The peptide (150 μg/mL) was prepared in either distilled H_2_O or 30% and 70% TFE (Aladdin, Shanghai, China) solutions. The spectra were measured at 25 °C by using a quartz cell with a 1mm light path. The mean residue molar ellipticity was calculated by using the equation as described [72].

### 4.3. Bacterial Strains

The antimicrobial activities of the peptides were tested against *S. aureus* ATCC29213, *E. faecalis* ATCC29212, *E. coli* ATCC25922, *K. pneumoniae* ATCC700603, *P. aeruginosa* ATCC27853 and *A. baumannii* ATCC19606. All these strains were purchased from the China Center of Type Culture Collection (CCTCC).

### 4.4. MIC Determination

The antimicrobial activities of the peptides were determined as previously described [26,73]. Briefly, the bacteria were incubated at 37 °C and 150 rpm until the log phase of growth. Next, the bacteria were diluted with Mueller-Hinton broth (MHB, Oxoid, UK) and prepared in a clear round-bottom polypropylene 96-well microtiter plate to achieve a final concentration of 5 × 10^5^ colony-forming unit (CFU)/mL. Twofold serial dilutions of the peptides were added into each well with the concentrations ranging from 0.625 to 40 μg/mL. The mixed peptides and cells were cultured at 37 °C and 150 rpm for 18 h, and the optical density (OD) was measured at 630 nm. The minimum inhibitory concentration was defined as the lowest peptide concentration with no detectable bacterial growth.

### 4.5. Hemolytic Activity Determination

The hemolytic activities of the peptides were determined against human red blood cells (hRBCs) [35,74]. Briefly, fresh hRBCs anticoagulated with sodium citrate were rinsed with 0.9% sodium chloride by centrifugation (1000× *g*, 5 min). Then, the cells were diluted to a final concentration of 2% (*v/v*) and prepared in a clear 96-well polypropylene microtiter plate. Next, the samples were mixed with a series of concentrations of the peptide (12.5, 25, 50, 100 and 200 μg/mL) and incubated at 37 °C for 1 h. After that, the samples were centrifuged (2000× *g*, 10 min), and the absorbances of the supernatants were measured at 540 nm to evaluate the release of hemoglobin. HRBCs treated with 1% Triton X-100 or 0.9% sodium chloride were taken as 100% and 0% hemolysis, respectively.

### 4.6. Cytotoxicity Determination

To determine the cytotoxicity of the peptide, the mouse fibroblast cell L929 (8000 cells per well) were seeded in a 96-well plate and incubated for 24 h in Dulbecco’s modified Eagle’s medium (DMEM, Gibco, USA) supplemented with 10% fetal bovine serum (FBS, Gibco, USA), 2 mM/L L-glutamine, 100 U/mL penicillin and 100 mg/mL streptomycin (1% P/S, Invitrogen, USA) at 37 °C in a 5% CO_2_ atmosphere. Then, a series of concentrations of the peptide (0, 5, 10, 20, 40, 60, 80 and 100 μg/mL) were added into each well and incubation was performed for 24 h. After that, the cytotoxicity of the peptide was measured with the CCK8 cell counting kit (Yeasen, Shanghai, China) by measuring the optical density at 450 nm [75].

### 4.7. Time–Killing Kinetics

The time–killing kinetics of the peptide against the different bacterial species were measured by the plate counting method [76]. Briefly, bacterial cells (2 × 10^6^ CFU/mL) were incubated with 1, 2 and 4 × MIC peptide at 37 °C and 150 rpm for 1 h. Cells without peptide treatment were used as a negative control. At each time interval (0, 5, 15, 30 and 60 min), aliquots of the samples were taken, diluted serially, and spread on Mueller-Hinton agar. The surviving bacterial colonies were counted after overnight incubation.

### 4.8. Membrane Permeability

Membrane permeability was evaluated by PI and SYTOX^TM^ Green uptake assays [47,48]. Both probes were purchased from Thermo Fisher (Invitrogen™, USA). Bacteria in the log phase of growth were collected (6000× *g*, 5 min) and washed three times with phosphate-buffered saline (PBS). Then, the bacteria (OD_630_ = 0.05) were mixed with PI (2 μM) or SYTOX^TM^ Green (30 nM), and incubated in a 96-well polypropylene microtiter plate for 5 min. After mixing with a series of concentrations of the peptide, fluorescence was recorded on a microplate reader (Molecular Devices SpectraMax i3x). The excitation and emission wavelengths were 503 nm and 530 nm for SYTOX^TM^ Green, and 535 nm and 617 nm for PI, respectively.

### 4.9. Cytoplasmic Membrane Depolarization

Membrane depolarization was monitored by using the cell membrane potential sensitive probe DiSC_3_-5 (Sigma, USA) [77,78]. Briefly, the bacterial cells were washed and diluted with 4-(2-hydroxyerhyl) piperazine-1-erhanesulfonic acid (HEPES), buffer (5 mM HEPES, 20 mM glucose, 100 mM potassium chloride, pH 7.4) to achieve a concentration of OD_630_ = 0.05, and prepared in a 96-well polypropylene microtiter plate. Then, the bacteria were mixed with 0.4 μM DiSC_3_-5 and incubated in the dark for 90 min to obtain a stable reduction in fluorescence. After that, the peptides were added to the sample and fluorescence was recorded immediately on a microplate reader (Molecular Devices SpectraMax i3x). The excitation wavelength was set at 622 nm and the emission wavelength was 670 nm.

## Figures and Tables

**Table 1:** Antimicrobial activity of Smp43(1-14).

Strains	MICs (μg/mL)
**Gram-positive bacteria**	
*Staphylococcus aureus* ATCC29213	5
*Enterococcus faecalis* ATCC29212	10
**Gram-negative bacteria**	
*Escherichia coli* ATCC25922	10
*Klebsiella pneumoniae* ATCC700603	10
*Pseudomonas aeruginosa* ATCC27853	10
*Acinetobacter baumannii* ATCC19606	5
HC_10_ ^a^	75
IC_10_ ^b^	92

^a^ HC_10_: the concentration of the peptide that causes 10% hemolysis of human red blood cells; ^b^ IC_10_: the concentration of the peptide that causes 10% inhibition of cell growth.

## Data Availability

Data will be available upon request from the author.

## Supplementary Materials

The following are available online at https://www.mdpi.com/article/10.3390/toxins13050343/s1, Table S1: Sequence alignments of scorpion venom-derived long-chain noncysteine-containing AMPs. Table S2: Amino acid sequences and physiochemical properties of Smp43-derived peptides. Table S3: Minimum inhibitory concentrations of Smp43-derived peptides against the tested bacterial strains. Table S4: Sequence alignments between Smp43(1-14) and other scorpion venom-derived short-chain noncysteine-containing AMPs.

## References

[1] Jeyaprakash A., Hoy M.A. (2008). First divergence time estimate of spiders, scorpions, mites and ticks (subphylum: Chelicerata) inferred from mitochondrial phylogeny. Exp. Appl. Acarol..

[2] Possani L.D., Becerril B., Delepierre M., Tytgat J. (1999). Scorpion toxins specific for Na^+^-channels. J. Biol. Inorg. Chem..

[3] De La Vega R.C.R., Schwartz E.F., Possani L.D. (2010). Mining on scorpion venom biodiversity. Toxicon.

[4] Chen Z.-Y., Hu Y.-T., Yang W.-S., He Y.-W., Feng J., Wang B., Zhao R.-M., Ding J.-P., Cao Z.-J., Li W.-X. (2012). Hg1, Novel Peptide Inhibitor Specific for Kv1.3 Channels from First Scorpion Kunitz-type Potassium Channel Toxin Family. J. Biol. Chem..

[5] Galvez A., Gimenez-Gallego G., Reuben J.P., Roy-Contancin L., Feigenbaum P., Kaczorowski G.J., Garcia M.L. (1990). Purification and characterization of a unique, potent, peptidyl probe for the high conductance calcium-activated potassium channel from venom of the scorpion *Buthus tamulus*. J. Biol. Chem..

[6] DeBin J., Strichartz G. (1991). Chloride channel inhibition by the venom of the scorpion *Leiurus quinquestriatus*. Toxicon.

[7] Debin J.A., Maggio J.E., Strichartz G.R. (1993). Purification and characterization of chlorotoxin, a chloride channel ligand from the venom of the scorpion. Am. J. Physiol. Physiol..

[8] Rowe A.H., Xiao Y., Rowe M.P., Cummins T.R., Zakon H.H. (2013). Voltage-Gated Sodium Channel in Grasshopper Mice Defends Against Bark Scorpion Toxin. Science.

[9] Chen Z., Wang B., Hu J., Yang W., Cao Z., Zhuo R., Li W., Wu Y. (2013). SjAPI, the First Functionally Characterized Ascaris-Type Protease Inhibitor from Animal Venoms. PLoS ONE.

[10] Liu H., Chen J., Wang X., Yan S., Xu Y., San M., Tang W., Yang F., Cao Z., Li W. (2015). Functional characterization of a new non-Kunitz serine protease inhibitor from the scorpion *Lychas mucronatus*. Int. J. Biol. Macromol..

[11] Zhu W., Gao H., Luo X., Ye X., Ding L., Hao J., Shu Z., Li S., Li J., Chen Z. (2020). Cloning and identification of a new multifunctional Ascaris-type peptide from the hemolymph of *Buthus martensii Karsch*. Toxicon.

[12] Ding L., Hao J., Luo X., Zhu W., Wu Z., Qian Y., Hu F., Liu T., Ruan X., Li S. (2018). The Kv1.3 channel-inhibitory toxin BF9 also displays anticoagulant activity via inhibition of factor XIa. Toxicon.

[13] Rashid M.H., Huq R., Tanner M.R., Chhabra S., Khoo K.K., Estrada R., Dhawan V., Chauhan S., Pennington M.W., Beeton C. (2015). A potent and Kv1.3-selective analogue of the scorpion toxin HsTX1 as a potential therapeutic for autoimmune diseases. Sci. Rep..

[14] Amirgholami N., Karampour N.S., Ghadiri A., Moghadam A.T., Dehcheshmeh M.G., Pipelzadeh M.H.A. (2020). crassicauda, *M*. *eupeus* and *H*. *lepturus* scorpion venoms initiate a strong in vivo anticancer immune response in CT26-tumor mice model. Toxicon.

[15] Cao L., Li Z., Zhang R., Wu Y., Li W., Cao Z. (2012). StCT2, a new antibacterial peptide characterized from the venom of the scorpion *Scorpiops tibetanus*. Peptides.

[16] Li Z., Hu P., Wu W., Wang Y. (2019). Peptides with therapeutic potential in the venom of the scorpion *Buthus martensii Karsch*. Peptides.

[17] Harrison P.L., Abdel-Rahman M.A., Miller K., Strong P.N. (2014). Antimicrobial peptides from scorpion venoms. Toxicon.

[18] Miyashita M., Kitanaka A., Yakio M., Yamazaki Y., Nakagawa Y., Miyagawa H. (2017). Complete de novo sequencing of antimicrobial peptides in the venom of the scorpion *Isometrus maculatus*. Toxicon.

[19] Corzo G., Escoubas P., Villegas E., Barnham K.J., He W., Norton R.S., Nakajima T. (2001). Characterization of unique amphipathic antimicrobial peptides from venom of the scorpion *Pandinus imperator*. Biochem. J..

[20] Gao B., Xu J., Rodriguez M.D.C., Lanz-Mendoza H., Hernández-Rivas R., Du W., Zhu S. (2010). Characterization of two linear cationic antimalarial peptides in the scorpion *Mesobuthus eupeus*. Biochimie.

[21] Wu S., Nie Y., Zeng X.-C., Cao H., Zhang L., Zhou L., Yang Y., Luo X., Liu Y. (2014). Genomic and functional characterization of three new venom peptides from the scorpion *Heterometrus spinifer*. Peptides.

[22] Nie Y., Zeng X.-C., Yang Y., Luo F., Luo X., Wu S., Zhang L., Zhou J. (2012). A novel class of antimicrobial peptides from the scorpion *Heterometrus spinifer*. Peptides.

[23] Dai L., Yasuda A., Naoki H., Corzo G., Andriantsiferana M., Nakajima T. (2001). IsCT, a Novel Cytotoxic Linear Peptide from Scorpion *Opisthacanthus madagascariensis*. Biochem. Biophys. Res. Commun..

[24] Dai L., Corzo G., Naoki H., Andriantsiferana M., Nakajima T. (2002). Purification, structure–function analysis, and molecular characterization of novel linear peptides from scorpion *Opisthacanthus madagascariensis*. Biochem. Biophys. Res. Commun..

[25] Yuan W., Cao L., Ma Y., Mao P., Wang W., Zhao R., Wu Y., Cao Z., Li W. (2010). Cloning and functional characterization of a new antimicrobial peptide gene StCT1 from the venom of the scorpion *Scorpiops tibetanus*. Peptides.

[26] Cao L., Dai C., Li Z., Fan Z., Song Y., Wu Y., Cao Z., Li W. (2012). Antibacterial Activity and Mechanism of a Scorpion Venom Peptide Derivative In Vitro and In Vivo. PLoS ONE.

[27] Zhao Z., Zhang K., Zhu W., Ye X., Ding L., Jiang H., Li F., Chen Z., Luo X. (2021). Two new cationic α-helical peptides identified from the venom gland of Liocheles australasiae possess antimicrobial activity against methicillin-resistant staphylococci. Toxicon.

[28] Ramírez-Carreto S., Quintero-Hernández V., Jiménez-Vargas J.M., Corzo G., Possani L.D., Becerril B., Ortiz E. (2012). Gene cloning and functional characterization of four novel antimicrobial-like peptides from scorpions of the family *Vaejovidae*. Peptides.

[29] Silva É.C., Camargos T.S., Maranhão A.Q., Silva-Pereira I., Silva L.P., Possani L.D., Schwartz E.F. (2009). Cloning and characterization of cDNA sequences encoding for new venom peptides of the Brazilian scorpion *Opisthacanthus cayaporum*. Toxicon.

[30] Gao B., Sherman P., Luo L., Bowie J., Zhu S. (2008). Structural and functional characterization of two genetically related meucin peptides highlights evolutionary divergence and convergence in antimicrobial peptides. FASEB J..

[31] Luna-Ramírez K., Quintero-Hernández V., Vargas-Jaimes L., Batista C.V., Winkel K.D., Possani L.D. (2013). Characterization of the venom from the Australian scorpion *Urodacus yaschenkoi*: Molecular mass analysis of components, cDNA sequences and peptides with antimicrobial activity. Toxicon.

[32] Luna-Ramirez K., Tonk M., Rahnamaeian M., Vilcinskas A. (2017). Bioactivity of Natural and Engineered Antimicrobial Peptides from Venom of the Scorpions *Urodacus yaschenkoi* and *U. manicatus*. Toxins.

[33] Zeng X.-C., Zhou L., Shi W., Luo X., Zhang L., Nie Y., Wang J., Wu S., Cao B., Cao H. (2013). Three new antimicrobial peptides from the scorpion *Pandinus imperator*. Peptides.

[34] Acevedo I.C.C., Silva P.I., Silva F.D., Araujo I., Alves F.L., Oliveira C.S., Oliveira V.X. (2019). IsCT-based analogs intending better biological activity. J. Pep. Sci..

[35] Li Z., Liu G., Meng L., Yu W., Xu X., Li W., Wu Y., Cao Z. (2016). K1K8: An Hp1404-derived antibacterial peptide. Appl. Microbiol. Biotechnol..

[36] Torres-Larios A., Gurrola G.B., Zamudio F.Z., Possani L.D. (2000). Hadrurin, a new antimicrobial peptide from the venom of the scorpion *Hadrurus aztecus*. J. Biol. Inorg. Chem..

[37] Zeng X.C., Li W.X., Peng F., Zhu Z.H. (2000). Cloning and characterization of a novel cDNA sequence encoding the precursor of a novel venom peptide (BmKbpp) related to a bradykinin-potentiating peptide from Chinese scorpion *Buthus martensii Karsch*. IUBMB Life.

[38] Moerman L., Bosteels S., Noppe W., Willems J., Clynen E., Schoofs L., Thevissen K., Tytgat J., Van Eldere J., Van Der Walt J. (2002). Antibacterial and antifungal properties of α-helical, cationic peptides in the venom of scorpions from southern Africa. J. Biol. Inorg. Chem..

[39] Miyashita M., Sakai A., Matsushita N., Hanai Y., Nakagawa Y., Miyagawa H. (2010). A Novel Amphipathic Linear Peptide with Both Insect Toxicity and Antimicrobial Activity from the Venom of the Scorpion *Isometrus maculatus*. Biosci. Biotechnol. Biochem..

[40] Hernández-Aponte C.A., Silva-Sanchez J., Quintero-Hernández V., Rodríguez-Romero A., Balderas C., Possani L.D., Gurrola G.B. (2011). Vejovine, a new antibiotic from the scorpion venom of *Vaejovis mexicanus*. Toxicon.

[41] Harrison P.L., Abdel-Rahman M.A., Strong P.N., Tawfik M.M., Miller K. (2016). Characterisation of three alpha-helical antimicrobial peptides from the venom of *Scorpio maurus palmatus*. Toxicon.

[42] Gautier R., Douguet D., Antonny B., Drin G. (2008). HELIQUEST: A web server to screen sequences with specific-helical properties. Bioinformatics.

[43] Pendleton J.N., Gorman S.P., Gilmore B.F. (2013). Clinical relevance of the ESKAPE pathogens. Expert Rev. Anti-Infect. Ther..

[44] Hu F., Zhu D., Wang F., Wang M. (2018). Current Status and Trends of Antibacterial Resistance in China. Clin. Infect. Dis..

[45] Yang J., Zhang Y. (2015). I-TASSER server: New development for protein structure and function predictions. Nucleic Acids Res..

[46] Schneider T., Kruse T., Wimmer R., Wiedemann I., Sass V., Pag U., Jansen A., Nielsen A.K., Mygind P.H., Raventós D.S. (2010). Plectasin, a Fungal Defensin, Targets the Bacterial Cell Wall Precursor Lipid II. Science.

[47] Mishra B., Narayana J.L., Lushnikova T., Wang X., Wang G. (2019). Low cationicity is important for systemic in vivo efficacy of database-derived peptides against drug-resistant Gram-positive pathogens. Proc. Natl. Acad. Sci. USA.

[48] Liu G., Yang F., Li F., Li Z., Lang Y., Shen B., Wu Y., Li W., Harrison P.L., Strong P.N. (2018). Therapeutic Potential of a Scorpion Venom-Derived Antimicrobial Peptide and Its Homologs Against Antibiotic-Resistant Gram-Positive Bacteria. Front. Microbiol..

[49] Bessalle R., Haas H., Goria A., Shalit I., Fridkin M. (1992). Augmentation of the antibacterial activity of magainin by positive-charge chain extension. Antimicrob. Agents Chemother..

[50] Ma Z., Yang J., Han J., Gao L., Liu H., Lu Z., Zhao H., Bie X. (2016). Insights into the Antimicrobial Activity and Cytotoxicity of Engineered α-Helical Peptide Amphiphiles. J. Med. Chem..

[51] Ghosh C., Sarkar P., Issa R., Haldar J. (2019). Alternatives to Conventional Antibiotics in the Era of Antimicrobial Resistance. Trends Microbiol..

[52] Fox J.L. (2013). Antimicrobial peptides stage a comeback. Nat. Biotechnol..

[53] Mookherjee N., Anderson M.A., Haagsman H.P., Davidson D.J. (2020). Antimicrobial host defence peptides: Functions and clinical potential. Nat. Rev. Drug Discov..

[54] Fjell C.D., Hiss J.A., Hancock R.E.W., Schneider G. (2011). Designing antimicrobial peptides: Form follows function. Nat. Rev. Drug Discov..

[55] Lee T.-H., Hall K.N., Aguilar M.-I. (2015). Antimicrobial Peptide Structure and Mechanism of Action: A Focus on the Role of Membrane Structure. Curr. Top. Med. Chem..

[56] Torres M.D.T., Sothiselvam S., Lu T.K., De La Fuente-Nunez C. (2019). Peptide Design Principles for Antimicrobial Applications. J. Mol. Biol..

[57] Juretić D., Simunić J. (2019). Design of α-helical antimicrobial peptides with a high selectivity index. Expert Opin. Drug Discov..

[58] Du Q., Hou X., Ge L., Li R., Zhou M., Wang H., Wang L., Wei M., Chen T., Shaw C. (2014). Cationicity-Enhanced Analogues of the Antimicrobial Peptides, AcrAP1 and AcrAP2, from the Venom of the Scorpion, *Androctonus crassicauda*, Display Potent Growth Modulation Effects on Human Cancer Cell Lines. Int. J. Biol. Sci..

[59] Guo X., Ma C., Du Q., Wei R., Wang L., Zhou M., Chen T., Shaw C. (2013). Two peptides, TsAP-1 and TsAP-2, from the venom of the Brazilian yellow scorpion, *Tityus serrulatus*: Evaluation of their antimicrobial and anticancer activities. Biochimie.

[60] Jiang Z., Vasil A.I., Hale J., Hancock R.E.W., Vasil M.L., Hodges R.S. (2009). Effects of net charge and the number of positively charged residues on the biological activity of amphipathic alpha-helical cationic antimicrobial peptides. Adv. Exp. Med. Biol..

[61] Jiang Z., Vasil A.I., Hale J.D., Hancock R.E.W., Vasil M.L., Hodges R.S. (2007). Effects of net charge and the number of positively charged residues on the biological activity of amphipathic α-helical cationic antimicrobial peptides. Biopolymers.

[62] Khara J.S., Lim F.K., Wang Y., Ke X.-Y., Voo Z.X., Yang Y.Y., Lakshminarayanan R., Ee P.L.R. (2015). Designing α-helical peptides with enhanced synergism and selectivity against *Mycobacterium smegmatis*: Discerning the role of hydrophobicity and helicity. Acta Biomater..

[63] Leptihn S., Har J.Y., Wohland T., Ding J.L. (2010). Correlation of Charge, Hydrophobicity, and Structure with Antimicrobial Activity of S1 and MIRIAM Peptides. Biochemie.

[64] Chen Y., Guarnieri M.T., Vasil A.I., Vasil M.L., Mant C.T., Hodges R.S. (2006). Role of Peptide Hydrophobicity in the Mechanism of Action of α-Helical Antimicrobial Peptides. Antimicrob. Agents Chemother..

[65] Strandberg E., Zerweck J., Horn D., Pritz G., Berditsch M., Bürck J., Wadhwani P., Ulrich A.S. (2015). Influence of hydrophobic residues on the activity of the antimicrobial peptide magainin 2 and its synergy with PGLa. J. Pept. Sci..

[66] Mihajlovic M., Lazaridis T. (2012). Charge distribution and imperfect amphipathicity affect pore formation by antimicrobial peptides. Biochim. Biophys. Acta Biomembr..

[67] Bessin Y., Saint N., Marri L., Marchini D., Molle G. (2004). Antibacterial activity and pore-forming properties of ceratotoxins: A mechanism of action based on the barrel stave model. Biochim. Biophys. Acta Biomembr..

[68] Yoneyama F., Imura Y., Ohno K., Zendo T., Nakayama J., Matsuzaki K., Sonomoto K. (2009). Peptide-Lipid Huge Toroidal Pore, a New Antimicrobial Mechanism Mediated by a Lactococcal Bacteriocin, Lacticin Q. Antimicrob. Agents Chemother..

[69] Järvå M., Lay F.T., Phan T.K., Humble C., Poon I.K.H., Bleackley M.R., Anderson M.A., Hulett M.D., Kvansakul M. (2018). X-ray structure of a carpet-like antimicrobial defensin-phospholipid membrane disruption complex. Nat. Commun..

[70] Colombo R. (1982). Solid-phase synthesis of chicken vasoactive intestinal peptide by a mild procedure using N alpha-9-fluorenylmethyloxycarbonyl amino acids. Int. J. Pep. Protein. Res..

[71] Fan Z., Cao L., He Y., Hu J., Di Z., Wu Y., Li W., Cao Z. (2011). Ctriporin, a New Anti-Methicillin-Resistant *Staphylococcus aureus* Peptide from the Venom of the Scorpion *Chaerilus tricostatus*. Antimicrob. Agents Chemother..

[72] Yang Z., He S., Wang J., Yang Y., Zhang L., Li Y., Shan A. (2019). Rational Design of Short Peptide Variants by Using Kunitzin-RE, an Amphibian-Derived Bioactivity Peptide, for Acquired Potent Broad-Spectrum Antimicrobial and Improved Therapeutic Potential of Commensalism Coinfection of Pathogens. J. Med. Chem..

[73] Mourtada R., Herce H.D., Yin D.J., Moroco J.A., Wales T.E., Engen J.R., Walensky L.D. (2019). Design of stapled antimicrobial peptides that are stable, nontoxic and kill antibiotic-resistant bacteria in mice. Nat. Biotechnol..

[74] Yin L.M., Edwards M.A., Li J., Yip C.M., Deber C.M. (2012). Roles of Hydrophobicity and Charge Distribution of Cationic Antimicrobial Peptides in Peptide-Membrane Interactions. J. Biol. Chem..

[75] Wu X., Wang Z., Li X., Fan Y., He G., Wan Y., Yu C., Tang J., Li M., Zhang X. (2014). In vitro and in vivo activities of antimicrobial peptides developed using an amino acid-based activity prediction method. Antimicrob. Agents Chemother..

[76] Shang D., Meng X., Zhang D., Kou Z. (2017). Antibacterial activity of chensinin-1b, a peptide with a random coil conformation, against multiple-drug-resistant *Pseudomonas aeruginosa*. Biochem. Pharmacol..

[77] Venkatesh M., Barathi V.A., Goh E.T.L., Anggara R., Fazil M.H.U.T., Ng A.J.Y., Harini S., Aung T.T., Fox S.J., Liu S. (2017). Antimicrobial Activity and Cell Selectivity of Synthetic and Biosynthetic Cationic Polymers. Antimicrob. Agents Chemother..

[78] Suzuki H., Wang Z.-Y., Yamakoshi M., Kobayashi M., Nozawa T. (2003). Probing the transmembrane potential of bacterial cells by voltage-sensitive dyes. Anal. Sci..

