# Supplementary material for: An Smp43-Derived Short-Chain α-Helical Peptide Displays a Unique Sequence and Possesses Antimicrobial Activity against Both Gram-Positive and Gram-Negative Bacteria
Source: Toxins (Basel). 2021 May 11;13(5):343. doi: 10.3390/toxins13050343 (PMC8150835; doi:10.3390/toxins13050343)
Supplement: Supplementary file 1 [file toxins-13-00343-s001.zip › toxins-1200955-supplementary proofdone.pdf]

Supplementary Materials: An Smp43-Derived Short-Chain α-Helical Peptide Displays a Unique Sequence and Possesses Antimicrobial Activity against Both Gram-Positive and Gram-Negative Bacteria

Xudong Luo, Li Ding, Xiangdong Ye, Wen Zhu, Kaiyue Zhang, Fangyan Li, Huiwen Jiang, Zhiwen Zhao and Zongyun Chen

Table S1. Sequence alignments of scorpion venom-derived long-chain noncysteine-containing AMPs.

Name	Sequence	AA[a]	Identity%	Reference
Smp43	G-VWDWIKKTAGKIWNSEPVKALKSQALNAAKNFVAEKIGATPS	43	100	[1]
Opistoporin-2	GKVWDWIKSTAKKLWNSEPVKELKNTALNAAKNFVAEKIGATPS	44	84.1	[2]
Opistoporin-1	GKVWDWIKSTAKKLWNSEPVKELKNTALNAAKNLVAEKIGATPS	44	81.8	[2]
Pandinin-1	GKVWDWIKSAAKKIWSSEPVSQLKGQVLNAAKNYVAEKIGATPT	44	75.0	[3]
Heterin-1	G-VWDWLKKTAKNVWNSDIVKQLKGKAINAAKNYVAEKIGATPS	43	74.4	[4]
Hadrurin	G-ILDTIKSIASKVWNSKTVQDLKRKGIN----WVANKLGVSPQAA	41	40.0	[5]
Vejovine	G-IWSSIKNLASKAWNSDIGQSLRNKAAGAINKFVADKIGVTPSQAAS	47	35.0	[6]
Im-1	-FSFKRLKGFAKKLWNSKLARKIRTKGLKYVKNFAKDMLSEGEEAPPAAEPPVEAPQ	56	21.4	[7]
Parabutoporin	---FK-LGSFLKKAWKSKLAKKLRAKGKEMLKDYAKGLLEGGSEEVPGQ	45	14.6	[2]
BmKbpp	---FR-FGSFLKKVWKSKLAKKLRSKGKQLLKDYANKVLNGPEEEAAAPAE	47	14.0	[8]

[a]AA, Number of residues.

Table S2. Amino acid sequences and physiochemical properties of Smp43-derived peptides.

	Name	Sequence	z[a]	<H>[b]	<μH>[c]	MW[d] (Da)
1	Smp43(1-14)	GVWDWIKKTAGKIW-NH_2	3	0.600	0.590	1687.0
2	Smp43(3-16)	WDWIKKTAGKIWNS-NH_2	3	0.467	0.572	1732.0
3	Smp43(5-18)	WIKKTAGKIWNSEP-NH_2	3	0.367	0.363	1656.9
4	Smp43(7-20)	KKTAGKIWNSEPVK-NH_2	4	0.094	0.206	1584.9
5	Smp43(9-22)	TAGKIWNSEPVKAL-NH_2	2	0.379	0.259	1512.8
6	Smp43(11-24)	GKIWNSEPVKALKS-NH_2	3	0.265	0.332	1555.8
7	Smp43(13-26)	IWNSEPVKALKSQA-NH_2	2	0.342	0.267	1569.8
8	Smp43(15-28)	NSEPVKALKSQALN-NH_2	2	0.131	0.231	1497.7
9	Smp43(17-30)	EPVKALKSQALNAA-NH_2	2	0.221	0.302	1438.7
10	Smp43(19-32)	VKALKSQALNAAKN-NH_2	4	0.102	0.312	1454.7
11	Smp43(21-34)	ALKSQALNAAKNFV-NH_2	3	0.301	0.327	1473.7
12	Smp43(23-36)	KSQALNAAKNFVAE-NH_2	2	0.134	0.273	1489.7
13	Smp43(25-38)	QALNAAKNFVAEKI-NH_2	2	0.265	0.334	1515.7
14	Smp43(27-40)	LNAAKNFVAEKIGA-NH_2	2	0.281	0.303	1444.7
15	Smp43(29-42)	AAKNFVAEKIGATP-NH_2	2	0.272	0.189	1415.6

[a]z, net charge; [b]<H>, the mean hydrophobicity value per residue; [c]<μH>, the hydrophobic moments. A value of 0.5 means that the peptide has about 50% of the maximum possible amphipathicity. [d]MW, the molecular weights of the peptides were measured by mass spectrometry.

Table S3. Minimum inhibitory concentrations of Smp43-derived peptides against the tested bacterial strains.

**Peptides**	**Smp43(1-14)**	**Smp43(3-16)**	**Smp43(5-18)**	**Smp43(7-20)**	**Smp43(9-22)**	**Smp43(11-24)**	**Smp43(13-26)**	**Smp43(15-28)**	**Smp43(17-30)**	**Smp43(19-32)**	**Smp43(21-34)**	**Smp43(23-36)**	**Smp43(25-38)**	**Smp43(27-40)**	**Smp43(29-42)**
**Bacterial strains**	Minimum inhibitory concentrations (μg/mL)														
**Gram-positive bacteria**															
Staphylococcus aureus ATCC29213	5	>40	>40	>40	>40	>40	>40	>40	>40	>40	>40	>40	>40	>40	>40
Enterococcus faecalis ATCC29212	10	>40	>40	>40	>40	>40	>40	>40	>40	>40	>40	>40	>40	>40	>40
**Gram-negative bacteria**															
Escherichia coli ATCC25922	10	>40	>40	>40	>40	>40	>40	>40	>40	>40	>40	>40	>40	>40	>40
Klebsiella pneumoniae ATCC700603	10	>40	>40	>40	>40	>40	>40	>40	>40	>40	>40	>40	>40	>40	>40
Pseudomonas aeruginosa ATCC27853	10	>40	>40	>40	>40	>40	>40	>40	>40	>40	>40	>40	>40	>40	>40
Acinetobacter baumannii ATCC19606	5	>40	>40	>40	>40	>40	>40	>40	>40	>40	>40	>40	>40	>40	>40
HC_{10}[a]	75	>200	>200	>200	>200	>200	>200	>200	>200	>200	>200	>200	>200	>200	>200

[a] HC_{10}, the concentration of the peptide that causes 10% hemolysis of of human red blood cells.

Table S4. Sequence alignments between Smp43(1-14) and other scorpion venom-derived short-chain noncysteine-containing AMPs.

Name	Sequence	AA[a]	Identity%	Reference
Smp43(1-14)	GVWDWIKKTAGKIW-NH_2	14	100	This study
Uy17	-ILSAIWSGIKGLL-NH_2	13	23.5	[9]
Uy192	-FLSTIWNGIKGLL-NH_2	13	23.5	[9]
IsCT	-ILGKIWEGIKSLF-NH_2	13	23.5	[10]
Spiniferin	-ILGEIWKGIKDIL-NH_2	13	23.5	[4]
BmKn2	-FIGAIARLLSKIF-NH_2	13	21.4	[11]
VsCT2	-FLKGIIDTVSKLF-NH_2	13	21.4	[12]
VsCT1	-FLKGIIDTVSNWL-NH_2	13	20.0	[12]
Um3	GFWGKLWEGVKSAI-NH_2	14	20.0	[9]
Smp13	-ILQDIWNGIKNLF-NH_2	13	17.7	[1]
UyCT3	-ILSAIWSGIKSLF-NH_2	13	17.7	[9]
VmCT1	-FLGALWNVAKSVF-NH_2	13	17.7	[12]
Pantinin-3	-FLSTIWNGIKSLL-NH_2	13	17.7	[13]
VmCT2	-FLSTLWNAAKSIF-NH_2	13	14.3	[12]
Hp1404	GILGKLWEGVKSIF-NH_2	14	14.3	[14]
UyCT1	GFWGKLWEGVKNAI-NH_2	14	14.3	[9]
UyCT5	-IWSAIWSGIKGLL-NH_2	13	14.3	[15]
Hp1035	-IFSAIGGFLKSIF-NH_2	13	14.3	[16]
Meucin-13	-IFGAIAGLLKNIF-NH_2	13	14.3	[17]
StCT1	GFWGSLWEGVKSVV-NH_2	14	14.3	[18]
StCT2	GFWGKLWEGVKSAI-NH_2	14	14.3	[19]
OcyC1	-ILSAIWSGIKSLF-NH_2	13	14.3	[20]
OcyC2	GILGKIWEGVKSLI-NH_2	14	14.3	[20]
Pantinin-1	GILGKLWEGFKSIV-NH_2	14	14.3	[13]
Pantinin-2	-IFGAIWKGISSLL-NH_2	13	14.3	[13]
Lausporin-1	-FLSALWGVAKSLF-NH_2	13	11.8	[21]
IsCT2	-IFGAIWNGIKSLF-NH_2	13	7.1	[10]
Um5	-IFKAIWGGIKSLF-NH_2	13	7.1	[9]
Hp1090	-IFKAIWSGIKSLF-NH_2	13	7.1	[16]
NDBP-5.5	-IFSAIAGLLSNLL-NH_2	13	7.1	[15]
Um4	-FFSALLSGIKSLF	13	0.00	[9]

[a]AA, Number of residues.

References

1. Harrison, P.L.; Abdel-Rahman, M.A.; Strong, P.N.; Tawfik, M.M.; Miller, K. Characterisation of three α-helical antimicrobial peptides from the venom of *Scorpio maurus palmatus*. *Toxicon.* **2016**, *117*, 30–36.
2. Moerman, L.; Bosteels, S.; Noppe, W.; Willems, J.; Clynen, E.; Schoofs, L.; Thevissen, K.; Tytgat, J.; Van Eldere, J.; Van Der Walt, J.; Verdonck, F. Antibacterial and antifungal properties of α-helical, cationic peptides in the venom of scorpions from southern Africa. *Eur J Biochem.* **2002**, *269*, 4799–4810.
3. Corzo, G.; Escoubas, P.; Villegas, E.; Barnham, K.J.; He, W.; Norton, R.S.; Nakajima, T. Characterization of unique amphipathic antimicrobial peptides from venom of the scorpion *Pandinus imperator*. *Biochem. J.* **2001**, *359*, 35–45.
4. Wu, S.; Nie, Y.; Zeng, X.C.; Cao, H.; Zhang, L.; Zhou, L.; Yang, Y.; Luo, X.; Liu, Y. Genomic and functional characterization of three new venom peptides from the scorpion *Heterometrus spinifer*. *Peptides.* **2014**, *53*, 30–41.

5. Torres-Larios, A.; Gurrola, G.B.; Zamudio, F.Z.; Possani, L.D. Hadrurin, a new antimicrobial peptide from the venom of the scorpion *Hadrurus aztecus*. *Eur. J. Biochem.* **2000**, *267*, 5023–5031.
6. Hernandez-Aponte, C.A.; Silva-Sanchez, J.; Quintero-Hernandez, V.; Rodriguez-Romero, A.; Balderas, C.; Possani, L.D.; Gurrola, G.B. Vejovine, a new antibiotic from the scorpion venom of *Vaejovis mexicanus*. *Toxicon.* **2011**, *57*, 84–92.
7. Miyashita, M.; Sakai, A.; Matsushita, N.; Hanai, Y.; Nakagawa, Y.; Miyagawa, H. A novel amphipathic linear peptide with both insect toxicity and antimicrobial activity from the venom of the scorpion *Isometrus maculatus*. *Biosci. Biotechnol. Biochem.* **2010**, *74*, 364–369.
8. Zeng, X.C.; Li, W.X.; Peng, F.; Zhu, Z.H. Cloning and characterization of a novel cDNA sequence encoding the precursor of a novel venom peptide (BmKbpp) related to a bradykinin-potentiating peptide from Chinese scorpion *Buthus martensii* Karsch. *IUBMB life* **2000**, *49*, 207–210.
9. Luna-Ramirez, K.; Tonk, M.; Rahnamaeian, M.; Vilcinskas, A. Bioactivity of natural and engineered antimicrobial peptides from venom of the scorpions *Urodacus yaschenkoi* and *U. manicatus*. *Toxins* **2017**, *9*, 22.
10. Dai, L.; Yasuda, A.; Naoki, H.; Corzo, G.; Andriantsiferana, M.; Nakajima, T. IsCT, a novel cytotoxic linear peptide from scorpion *Opisthacanthus madagascariensis*. *Biochem. Biophys. Res. Commun.* **2001**, *286*, 820–825.
11. Cao, L.; Dai, C.; Li, Z.; Fan, Z.; Song, Y.; Wu, Y.; Cao, Z.; Li, W. Antibacterial activity and mechanism of a scorpion venom peptide derivative in vitro and in vivo. *PLoS ONE* **2012,** 7, e40135.
12. Ramirez-Carreto, S.; Quintero-Hernandez, V.; Jimenez-Vargas, J.M.; Corzo, G.; Possani, L.D.; Becerril, B.; Ortiz, E. Gene cloning and functional characterization of four novel antimicrobial-like peptides from scorpions of the family Vaejovidae. *Peptides* **2012**, *34*, 290–295.
13. Zeng, X.C.; Zhou, L.; Shi, W.; Luo, X.; Zhang, L.; Nie, Y.; Wang, J.; Wu, S.; Cao, B.; Cao, H. Three new antimicrobial peptides from the scorpion *Pandinus imperator*. *Peptides* **2013**, *45*, 28–34.
14. Li, Z.; Xu, X.; Meng, L.; Zhang, Q.; Cao, L.; Li, W.; Wu, Y.; Cao, Z. Hp1404, a new antimicrobial peptide from the scorpion *Heterometrus petersii*. *PLoS ONE* **2014,** 9, e97539.
15. Trentini, M.M.; das Neves, R.C.; Santos, B.P.; DaSilva, R.A.; de Souza, A.C.; Mortari, M.R.; Schwartz, E.F.; Kipnis, A.; Junqueira-Kipnis, A.P. Non-disulfide-bridge peptide 5.5 from the scorpion *Hadrurus gertschi* inhibits the growth of *Mycobacterium abscessus subsp. massiliense*. *Front. Microbiol.* **2017**, *8*, 273.
16. Yan, R.; Zhao, Z.; He, Y.; Wu, L.; Cai, D.; Hong, W.; Wu, Y.; Cao, Z.; Zheng, C.; Li, W. A new natural α-helical peptide from the venom of the scorpion *Heterometrus petersii* kills HCV. *Peptides* **2011**, *32*, 11–19.
17. Gao, B.; Sherman, P.; Luo, L.; Bowie, J.; Zhu, S. Structural and functional characterization of two genetically related meucin peptides highlights evolutionary divergence and convergence in antimicrobial peptides. *FASEB. J.* **2009**, *23*, 1230–1245.
18. Yuan, W.; Cao, L.; Ma, Y.; Mao, P.; Wang, W.; Zhao, R.; Wu, Y.; Cao, Z.; Li, W. Cloning and functional characterization of a new antimicrobial peptide gene StCT1 from the venom of the scorpion *Scorpiops tibetanus*. *Peptides.* **2010**, *31*, 22–26.
19. Cao, L.; Li, Z.; Zhang, R.; Wu, Y.; Li, W.; Cao, Z. StCT2, a new antibacterial peptide characterized from the venom of the scorpion *Scorpiops tibetanus*. *Peptides* **2012**, *36*, 213–220.
20. Silva, E.C.; Camargos, T.S.; Maranhao, A.Q.; Silva-Pereira, I.; Silva, L.P.; Possani, L.D.; Schwartz, E.F. Cloning and characterization of cDNA sequences encoding for new venom peptides of the Brazilian scorpion *Opisthacanthus cayaporum*. *Toxicon.* **2009**, *54*, 252–261.
21. Zhao, Z.; Zhang, K.; Zhu,W.; Ye, X.; Ding, Li.; Jiang, H.; Li, F.; Chen, Z.; Luo, X. Two new cationic α-helical peptides iden-tified from the venom gland of *Liocheles australasiae* possess antimicrobial activity against methicillin-resistant staphylococci, *Toxicon*, **2021**, In Press, https://doi.org/10.1016/j.toxicon.2021.04.002.
